# Trait Hostility, Perceived Stress, and Sleep Quality in a Sample of Normal Sleepers

**DOI:** 10.1155/2013/735812

**Published:** 2013-04-22

**Authors:** Nicholas D. Taylor, Gary D. Fireman, Ross Levin

**Affiliations:** ^1^Psychology Department, Suffolk University, 41 Temple Street, Boston, MA 02114, USA; ^2^Independent Practice, 25 West 86th Street No. 3, New York, NY 10024, USA

## Abstract

*Objective*. To date, no studies have directly examined the effects of cognitive trait hostility on prospectively assessed sleep quality. This is important as individuals with heightened trait hostility demonstrate similar patterns of reactivity to perceived stressors as is often reported by poor sleepers. The present study hypothesized that increased trait hostility is associated with poorer subjective sleep quality and that perceived stress mediates this relationship. *Methods*. A sample of 66 normal sleepers completed daily sleep and stress logs for two weeks. Trait hostility was measured retrospectively. *Results*. The cognitive dimension of trait hostility was significantly correlated with subjectively rated sleep quality indicators, and these relationships were significantly mediated by perceived daily stress. Individuals with higher levels of trait cognitive hostility reported increased levels of perceived stress which accounted for their poorer sleep ratings as measured by both retrospective and prospective measures. *Conclusions*. Overall, the findings indicate that high levels of cognitive hostility are a significant risk factor for disturbed sleep and suggest that this might be a fruitful target for clinical intervention.

## 1. Introduction

The relationship between trait hostility and sleep quality remains underexplored despite the empirical indications that individuals with increased hostility experience more stress, a factor known to degrade sleep quality [1–4]. Despite its widespread use as an outcome variable, there is no standard definition for sleep quality. Investigators frequently use both objective measures, such as total sleep duration, efficiency, and sleep onset latency, as well as subjective self-report measures to assess sleep quality [5]. While a number of variables can influence one's day-to-day sleep outcomes, stress is strongly associated with sleep disruption [1, 6]. Stress is thought to act on sleep primarily via increased cognitive and somatic arousal during the presleep period [7]. In a recent study, Morin et al. [6] confirmed that hyperarousal during the presleep period mediates the relationship between stress and sleep quality in normal and disordered sleepers alike. The study suggests that individual variation in stress reactivity determines the extent to which stress degrades sleep quality. Consistent with this finding, studies have demonstrated that poor sleepers are characteristically hyperreactive to stress [1, 2, 6, 8].

 Pronounced stress reactions are characteristic of individuals who score highly on measures of trait hostility [3]. The trait hostility construct is organized into three major components: cognitive, affective, and behavioral [9]. The cognitive components, cynicism and hostile attribution, reflect the extent to which negative beliefs about others are held and a tendency to interpret the antagonistic behavior of others as expressly directed at the self. The affective component of hostility consists of the tendency to experience several negative emotions including anger, annoyance, resentment, disgust, and contempt. The behavioral component of hostility reflects an individual's tendency to act aggressively. While the three components of hostility are interrelated, one does not necessitate the presence of the other, and measures of hostility that incorporate all three components often find only moderate intercorrelations [9].

Numerous studies [3, 10, 11] have found that individuals who report higher levels of trait hostility are highly reactive to and slower to recover from interpersonal stress. Although preliminary evidence suggests that individuals high in trait hostility report poorer sleep than controls [12–14], few studies have directly examined the relationship between hostility, stress, and sleep outcomes. The current study builds upon these preliminary findings by testing trait hostility's association with measures of retrospective and prospective sleep quality. Additionally, the current study examines whether the relationship between hostility and sleep quality is mediated by increased sensitivity to stress. 

Stress is perhaps, the most studied psychosocial precipitant of sleep disturbance. Current models describe stress as a process with four basic aspects: the stress stimuli, perceived stress, the stress response (physiological, affective, and cognitive), and feedback from the stress response [15]. Thus, there are multiple ways in which stress may be measured, many of which address only a portion of the construct.

Observational and experimental studies link stress exposure to sleep quality, and it is widely acknowledged that stress plays a causal role in initiating sleep disruption and the onset of disordered sleeping [1, 6, 16]. However, the relationship between stress and sleep is complicated by individual variability in how intensely one experiences and responds to stress [2, 17]. The cognitive model of insomnia [7] emphasizes that some individuals are more prone to sleep disturbance and insomnia than others because of this variability in stress responding. In this model, individuals who are more affected by stress stimuli struggle with sleep because they have stronger or more frequent stress reactions and are more likely to engage in behaviors that exacerbate the impact of a stressor beyond the initial stress response, such as rumination or worry. The consequence is increased presleep arousal that is incompatible with sleep onset [7].

Morin et al. [6] tested this component of the cognitive model of insomnia. Participants completed measures of daily stress experience, presleep arousal, and sleep quality over the course of 21 days. Increased perceived stress was negatively associated with sleep quality across participants; however, poor sleepers were much more sensitive to stress events compared to normal sleepers. Poor sleepers rated major life events and daily stressors as more intense and more disruptive to their functioning than good sleepers. Further, presleep arousal mediated the relationship between stress experience and subsequent sleep quality. Consistent with the finding that poor sleepers are hyperaroused during the presleep period, poor sleepers also demonstrate signs of hyperarousal during the day [18, 19]. The findings suggest that groups that are characteristically sensitive to or more reactive to stress may also be at increased risk of sleep disturbance.

Individuals who score high on measures of trait hostility share a striking number of characteristics with poor sleepers, such as pronounced reactions to stress, increased negative affect, and ruminative tendencies which can prolong arousal following stress [8, 14, 20]. Further, trait hostility has been widely studied in connection to other outcomes related to pronounced stress and arousal, such as cardiovascular disease [4, 21, 22]. Such findings suggest that trait hostile individuals may be particularly vulnerable to stress-related-sleep disruption and the widespread consequences of poor sleep.

The psychophysiological reactivity model hypothesizes that hostile individuals experience anger more intensely and for longer periods than controls, causing more sustained and more intense activation of the sympathetic nervous system [23]. Additionally, hostility is associated with rumination, a likely mechanism through which daytime stress reactions are extended into the presleep period [24]. Empirical findings generally support the hyperreactivity hypothesis. The studies indicate that higher levels of hostility are associated with exaggerated cardiovascular reactivity (CVR) to stress [3, 11, 25] and that hostile individuals also take longer to recover from stressors compared to controls [26]. As such, a significant body of literature links hostility to pronounced stress reactions and pronounced stress reactions to poor sleep. Further, multiple studies have found associations between the behavioral and affective components of the hostility construct and sleep outcomes [10, 12, 27, 28]. Despite the relevance of stress to both sleep and hostility, research incorporating all three variables is virtually nonexistent.

To date, only one study has simultaneously examined sleep quality, hostility, and stress. Brissette and Cohen [14] examined a community sample of 47 adults over the course of 7 days. Across participants, increased interpersonal stress predicted to higher reported negative affect. This effect, however, was pronounced among individuals high in cynical hostility meaning they were more significantly impacted by conflict than other participants. Further, on days in which individuals experienced conflict, between-person differences in cynical hostility predicted the impact conflict had on sleep. This is consistent with the proposal that trait hostility influences individual reactivity to interpersonal stress, which subsequently impacts sleep quality. However, this study appeared to conflate self-report sleep duration with sleep quality and did not use a validated measure of stress, making the findings difficult to interpret and in need of replication.

Models of sleep disturbance emphasize the importance of individual differences in stress responding [7]. If individuals high in trait hostility are more reactive to stress than controls, and stress exposure is an established factor that degrades sleep quality, it follows that hostile individuals are likely to have poorer sleep compared to controls. Differences in stress responding likely mediate the relationship between trait hostility and sleep quality. This study tests these potential relationships. We hypothesize that self-reported daily sleep quality will be negatively associated with trait hostility such that poorer sleep quality will be associated with increased hostility. Second, we predict that perceived stress will be positively associated with trait hostility such that increased trait hostility will predict to increases in perceived stress. Third, we predict that stress exposure will be negatively associated with measures of sleep quality such that increased stress will predict reduced sleep quality. Finally, we predict that perceived stress will mediate any relationship between trait hostility and sleep quality.

A number of methodological improvements in the current study will extend previous work in this area. Specifically, this study utilizes multiple subscales of the Cook-Medley Hostility Scale [29] rather than the cynicism scale alone, allowing for a comparison of the cognitive components of hostility (cynicism, hostile attribution) and the behavioral component of hostility (aggressive responding). This study also utilized cross-sectional and prospective design elements over an extended time period (14 consecutive nights) to obtain more reliable estimates of perceived stress and sleep quality.

## 2. Method

### 2.1. Participants

A convenience sample of 73 undergraduate psychology students (26 men, 47 women) aged from 17 to 25 years (*M* = 19.04, SD = 1.57) was recruited from a university in the Northeast with 56 self identified as Caucasian, 3 as African American, 4 as Hispanic, 4 as Asian, 3 as mixed ethnicity, and 3 as Other Ethnicity. Individuals interested in participating signed up for the study via sign-up sheets placed in the psychology department of the university. Candidates were then contacted to ensure that they met inclusion criteria and to schedule a time to complete the initial surveys. The sample was intended to include a range of sleepers in order to generalize to the larger community, so only individuals that had a known condition affecting heart function or blood pressure, who took a medication that interfered with stress reactivity (such as benzodiazepines), or who used drugs or alcohol daily were excluded from participation. Of the original sample, 7 individuals failed to complete a minimum of ten daily surveys over the course of a two-week reporting period and were excluded from analysis for lack of sufficient data. There were no significant differences between individuals who completed the study and those lost to attrition. 92.4% of the sample completed 12 or more surveys out of a possible 14. The final sample consisted of 66 participants. 

### 2.2. Cross-Sectional Measures

#### 2.2.1. Demographics

This survey was created for the purposes of this investigation. It includes questions about gender, ethnic identification, living arrangements, a variety of health questions, and other health variables known to influence cardiovascular function and arousal.

#### 2.2.2. Pittsburgh Sleep Quality Index (PSQI)

The PSQI is one of the most widely used measures of subjective sleep quality. The PSQI is a retrospective, self-report inventory that asks participants to report on their subjectively experienced sleep quality and disturbances over the last month [30]. The index is composed of 19 items which compose seven component scores: subjective sleep quality, sleep latency, sleep duration, habitual sleep efficiency, sleep disturbances, use of sleep medication, and daytime dysfunction. A global score is derived by combining the composite scores and is the primary retrospective sleep quality variable used in our analysis. The index was normed with both good and poor sleepers that were slightly older than our sample (20–40 years old) and has proven useful as a clinical tool for identifying poor sleepers. Using a criterion score of five to identify poor sleepers, the sensitivity of the measure was 89.6% and specificity was reported at 86.5%  (kappa = .75) [30]. In the current sample, the average global PSQI score was 7.0  (SD = 3.13, range of 2 to 14). The scale was reverse coded for analyses to ease interpretation such that higher scores indicate better sleep.

#### 2.2.3. Perceived Stress Scale (PSS)

The PSS is a retrospective, self report measure of stress appraisal that asks about participants perceptions of stress over the previous month [31]. The scale has ten items from which a single perceived stress score is derived. This value is the primary indicator of retrospective perceived stress used in this study. Studies reporting psychometrics for the scale indicate good internal consistency (*α* = .84–.86) in various samples and good test-retest reliability in a college sample (*r* = .85) [31]. The scale also demonstrates good discriminant validity with a measure of depression and daily life stress [32]. In the current sample, the scale displayed adequate internal reliability (*α* = .88). 

#### 2.2.4. Cook-Medley Hostility Scale (CMHo)

The CMHo Scale is a widely used 50 item self-report measure of trait hostility [29] with good discriminant and convergent validity [9, 33]. Factor analysis [9] revealed six subsets of items the authors categorized as cynicism, hostile attribution, aggressive responding, hostile affect, social avoidance, and others. Barefoot et al. [9] describes the measure as primarily cognitive, with some behavioral and affective loading items. This study used the cynicism and hostile attribution item subsets to reflect the cognitive component of the trait hostility construct and the aggressive responding item subset as an indication of the behavioral component of hostility. The other subscales were dropped to limit time burden. To identify a reliable subset of questions within the behavioral scale, a principal component analysis was completed and two components were retained. The component with the best reliability was composed of 4 questions (*α* = .5) and was utilized in our analyses.

### 2.3. Prospective Self-Report Measures

#### 2.3.1. The Sleep/Dream Checklist (SDC)

The SDC, a 21 item self-report log, was developed by Levin and Fireman [34] to track various aspects of sleep quality and experience over time. It includes questions about total sleep time, sleep efficiency, sleep quality, disturbed dreaming, and affect. The measure has been associated with the Symptom Checklist-90-Revised, the State-Trait Anxiety Inventory, and the Beck Depression Inventory indicating good predictive validity [34].

Prospective daily sleep quality (DSQ) was measured by asking participants “What was the quality of your sleep last night?” Participants responded using a 9-point Likert scale. All daily ratings were averaged across the two-week reporting period for use in analyses.

#### 2.3.2. Daily Stress Inventory (DSI)

The DSI is a prospective, daily measure of the individualized impact of relatively minor stress events [35]. Participants indicate whether each of 58 events occurred for them within the preceding 24-hour period and then provide a severity rating for each event that occurred. For example, the inventory lists “Competed with someone” and “Criticized or verbally attacked” as possible events and asks participants to either mark that it did not occur or rate the severity of the stressor on a seven point Likert scale ranging from “1 = occurred but was not stressful” to “7 = caused me to panic.” The measure has good convergent and divergent validity with other measures of stress and mood as well as sound internal validity (*α* = .83 to .87) [35].

To decrease the daily time commitment for participants, 19 items were eliminated that were likely to have a low occurrence among an undergraduate sample. The final measure included 39 events and two blank spaces for additional write-in events. The measure outcomes include the number of events that are endorsed as having occurred (stress events per day) and the sum total of the impact rating of these events (stress per day). These were used to calculate the average impact rating an individual endorses when experiencing a stress event (stress per event), our prospective estimate of perceived stress. 

### 2.4. Procedure

 The current study's design is correlation based and employed both cross-sectional and prospective measures [36]. Data collection occurred in two phases. Following recruitment, participants attended an initial 20-minute survey session. After informed consent was obtained, a brief demographic questionnaire designed for this study was completed, as well as the Pittsburgh Sleep Quality Index [30], Perceived Stress Scale [31], and the cynicism, hostile attribution, and aggressive responding subscales of the Cook-Medley Hostility Scale [9, 29]. Upon completing the initial surveys, participants were given instructions regarding the format and completion of the daily online surveys. Participants then completed the sleep/dream checklist [34] and the Daily Stress Inventory [35] online, daily, for two weeks. Participants received an email containing an internet link to that day's survey each day. The surveys were administered using an online hosting service (http://www.keysurvey.com/). 

Data analyses were completing using a popular statistical software package. Initial analyses focused on establishing the psychometric properties of the collected data set. Internal consistency for measures was calculated using Cronbach's alpha. Variables were also examined to ensure they met assumptions of any statistical tests for which they were utilized. A series of Pearson's correlations describe the relationship between retrospective and prospective measures of sleep and stress to establish convergent validity. Convergent validity was also examined by replicating previously found associations between relevant variables. Examples include a positive association between cognitive hostility and some stress variables and negative associations between stress variables and sleep quality. A series of Pearson's correlations and regression analyses were used to test the studies main hypotheses. All mediation analyses were conducted using the process outlined in Baron and Kenny [37].

## 3. Results

### 3.1. Trait Hostility and Sleep Quality

 The relationship between trait hostility and sleep quality was initially investigated using a series of Pearson's product-moment correlation coefficients. Cognitive hostility (as measured by the cynicism and hostile attribution subscales of the CMHo Scale) and behavioral hostility (as measured by the Aggressive Responding subscale) were correlated with the sleep quality component scores from the PSQI and the SDC. 

Significant negative correlations were found between cognitive hostility and both measures of sleep quality such that increased hostility related to decreases in both retrospective sleep quality (*r*(66) = −.43, *P* < .001) and prospectively measured sleep quality (*r*(66) = −.26, *P* < .05). Behavioral hostility showed no meaningful association with the sleep quality variables. 

### 3.2. Trait Hostility and Perceived Stress

The relationship between trait hostility and the three stress variables was investigated using Pearson's correlations. The cognitive and behavioral subscales of the Cook-Medley Hostility Scale were compared with a measure of retrospective perceived stress, the PSS, as well as the primary measures from the DSI: stress impact per event and stress events per day. There was a positive correlation between trait cognitive hostility and prospectively measured perceived stress, (*r*(66) = .32, *P* < .01), as well as retrospective perceived stress (*r*(66) = .36, *P* < .01). No relationship was found between cognitive hostility and the number of stress events experienced per day. No relationship was found between behavioral hostility and the stress measures.

### 3.3. Sleep Quality and Perceived Stress

#### 3.3.1. Retrospective Sleep Quality

The sleep quality and stress variables were also compared using a series of Pearson's correlations. There was a strong negative correlation between the PSQI and PSS (*r*(66) = −.584, *P* < .001) such that poorer sleep quality was associated with more perceived stress. PSQI scores were also moderately associated with the tendency to rate stress events as more severe (*r*(66) = −.432, *P* < .001). The PSQI did not significantly relate to the average frequency of stress events per day.

#### 3.3.2. Prospective Sleep Quality

Prospective measures of sleep were also associated with levels of daily and retrospective perceived stress such that increased stress experience predicted poorer sleep. DSQ was strongly associated with the average severity ratings per stress event (*r*(66) = −.52, *P* < .001) and scores on the PSS (*r*(66) = −.52, *P* < .001). However, daily sleep quality was not significantly correlated with the frequency of stress events per day. 

### 3.4. Perceived Stress Mediates the Relationship between Hostility and Sleep Quality

To test our hypothesis that perceived stress mediates the relationship between trait hostility and self-reported sleep quality, a series of regression analyses were run using the procedure outlined by Baron and Kenny [37] as well as a bootstrapping method that tests for indirect effects.

#### 3.4.1. Cognitive Hostility, Average Stress per Event, and Daily Sleep Quality

The first model examined whether the relationship between cognitive hostility and DSQ was mediated by the tendency to rate daily stress events as more severe. As can be seen in Table 1, cognitive hostility was significantly correlated with daily sleep quality. A series of regression analyses were employed to investigate average stress as a possible mediator. As shown in Table 2, the inclusion of the stress variable reduced the variance cognitive hostility explained in DSQ from 6.8%  (*sr*⁡^2^ = .068) in the initial model to 1.0%  (*sr*⁡^2^ = .010) in the final model. Bias-corrected confidence intervals further supported a significant indirect effect via stress per event (standardized indirect effect = −.0550, *P* < .001, 95% CI = −.108; −.013). The tendency to rate stress events as more severe partially mediated the relationship between cognitive hostility and daily subjective sleep quality.

#### 3.4.2. Cognitive Hostility, Perceived Stress, and Retrospective Sleep Quality

The second mediation analysis examined whether the relationship between cognitive hostility and retrospective sleep quality (PSQI) is mediated by retrospective perceived stress. As seen in Table 3, the model was significant, with the PSS accounting for 37.4% of the variance in the PSQI. The addition of the mediator variable attenuated the impact of cognitive hostility on the IV, though hostility remained a significant predictor of the PSQI. The inclusion of the perceived stress variable reduced the amount of variance cognitive hostility explained in the PSQI from 17.7%  (*sr*⁡^2^ = .177) in the first model to 5.3%  (*sr*⁡^2^ = .053) in the final model. Bias-corrected confidence intervals further supported a significant indirect effect via perceived stress (standardized indirect effect = .1613, *P* < .001, 95% CI= .072; .279). Perceived stress partially mediated the relationship between cognitive hostility and sleep quality. 

## 4. Discussion

The present study found that increased trait hostility is associated with decreased retrospective and prospectively measured sleep quality and that this relationship is significantly mediated by one's response to stress. Significantly, only the cognitive component of hostility was associated with heightened stress and sleep quality. This is consistent with previous studies which found differential associations between the components of hostility and both stress and sleep. For instance, Wilkinson [38] found no association between the aggressive responding subscale of the Cook-Medley Hostility Scale and coronary heart disease but did find the cynicism subscale to be predictive of health outcomes. Similarly, Ireland and Culpin [13] found that a measure of cynicism was a much stronger predictor of sleep quality than a measure of aggression.

Individuals who scored highly on cognitive hostility reported more daily and retrospective perceived stress compared to participants who scored low on the subscale. Importantly, while hostility was unrelated to stress event frequency, high hostility subjects reported heightened reactivity to their stressors and rated their stress as more severe than low hostile participants. This is consistent with Williams et al. [23] psychophysiological reactivity model, which predicts more intense cognitive and somatic reactions to stress events for high hostile individuals. The finding is also consistent with studies documenting hyperreactivity in hostile participants using measures of cardiovascular arousal [3, 10, 11].

Our study replicated previous findings associating increased stress with poor sleep quality [6, 18, 19, 39] utilizing both prospective and retrospective measures of stress and sleep. Generally, increased stress was associated with poorer sleep outcomes. Notably, just as stress event frequency did not relate to hostility, event frequency did not significantly relate to any sleep quality measure.

The subjective measures of sleep quality had a consistently strong, negative association with perceived stress. These data support earlier findings by Morin et al. [6] in which insomniacs and controls reported a similar number of stressful events over 21 days, but differed in their response to stress in that insomniacs rated their events as more intense and impactful. In addition, the data support earlier findings by Healey et al. [40] and Sadeh et al. [2] indicating that higher levels of perceived stress following a life event or lab induced stressor were associated with poorer subjective sleep quality.

The studies primary hypothesis was that increased reactivity to stress would account for a significant portion of the relationship between hostility and sleep quality. Consistent with the hypothesis, mediation analyses indicated that hostility is related to sleep primarily via increases in stress experience. This finding was true for both prospective and retrospectively measured sleep quality and perceived stress and indicates that trait hostility is a risk factor for stress-related sleep disruption.

This hypothesis was primarily based on two theories. The first theory, the psychophysiological reactivity model, asserts that individuals high in hostility are cognitively and somatically hyperreactive to stress [23]. The cognitive model of insomnia proposes that cognitive and somatic hyperarousal following stress is a major pathway through which sleep is degraded [7]. Together, the two models suggest that trait hostility is a possible risk factor for poor sleep to the extent that hostility results in increased arousal following stress. The current results are generally consistent with this logic in that more cognitive hostility was associated with poorer sleep quality by way of increased perceived stress. Previous studies have shown that perceived stress relates to sleep quality through increased cognitive and somatic arousal during the presleep period [6].

One possible explanation for these findings is that individuals high in cognitive hostility attend to and ruminate more on their internal responses to stress. The cognitive model of insomnia prioritizes this type of repetitive, negatively toned cognitive activity as a major pathway through which sleep can be disrupted [7]. These types of behaviors can result in a stronger cognitive and somatic response to stress and heightened presleep arousal, reducing sleep quality [7, 8]. Future studies should examine rumination and pre-sleep arousal as possible mechanisms through which stress acts on sleep in this population.

While the behavioral component of trait hostility was unrelated to sleep quality, cognitive hostility was associated with subjective sleep quality. Previous studies have found similar associations [12–14]. In the current study, the strength of the association varied between sleep indicators. The PSQI for instance was moderately correlated with cognitive hostility while participant's daily ratings of their sleep quality were less closely associated with cognitive hostility.

Overall, the findings support our hypothesis that heightened trait hostility acts as a risk factor for poor sleep. However, behavioral hostility was not associated with any measure of sleep quality. Previous studies exploring aggression and sleep have generally reported a negative association between these two variables [13, 27, 28, 41, 42]. Interestingly, these studies looked primarily at objective measures of sleep such as sleep duration or the presence of a significant sleep disorder. Subjective sleep quality was not directly examined. In addition, these studies all utilized samples (incarcerated juveniles, adult sex offenders, children between the age of 2 and 14, clinically disturbed sleepers, and adolescent substance abusers) which may not be representative of the broader population. These measurement and sample differences may help clarify our null findings.

Causality cannot be directly addressed in the present study. The question of whether increased hostility leads to poor sleep, is caused by poor sleep, or some combination of the two will have to be resolved using a design appropriate for establishing causality. For instance, future investigators may attempt to manipulate levels of hostility and measure any subsequent changes in sleep quality. Studies already exist in which interventions targeting sleep quality impact variables associated with the hostility construct, such as aggression [14].

An interesting possibility is that the relationship between hostility and sleep is reciprocal. Trait hostility may actively degrade sleep via increased arousal as we suspect, and poor sleep may exacerbate hostile responding and stress responses. Conversely, good sleep might serve to diminish hostility, even in individuals who are high in trait hostility. Good sleep may therefore minimize the likelihood of negative outcomes associated with high trait hostility, such as coronary heart disease. Additionally, if arousal proves to be an important mechanism through which cognitive hostility impacts sleep and degrades health, then there are multiple points at which that process might be disrupted through intervention. One could actively target cognitive hostility or perceived stress through counseling.

A number of methodological and design issues in the present study suggest caution in interpreting our findings. The current study utilized self-report measures of hostility, stress, and sleep, which raises concerns of biased responding and shared method variance. Objective measures of sleep, hostility, and stress would be valuable supplements to any self-report instruments utilized in future studies. Additionally, the current study had a relatively small sample size consisting primarily of young, Caucasian college students. Future studies in this area should utilize broader samples. Last, we did not directly measure presleep arousal or utilize a design that allowed us to establish directionality. Important extensions of any subsequent studies will be to explicitly test for presleep arousal levels, establish causality, and look for the presence of proposed causal mechanisms such as rumination and possible moderators such as coping style.

Our findings suggest a number of fruitful avenues for clinical intervention. Interventions targeting at reducing hostile cognitions and behaviors thought to exacerbate stress responding in this group, such as rumination, might diminish the impact of hostility on sleep quality and health. Conversely, behavioral sleep interventions are generally low risk and highly effective. If poor sleep quality does exacerbate hostility, improving sleep quality might help to minimize some of the negative social and health effects trait hostility has been linked to. In conclusion, the current study provides evidence that increased trait cognitive hostility is associated with poorer subjective sleep via increases in perceived stress. Additional work is required to address issues of causality and directionality, as there are possible implications for the treatment and health of both poor sleepers and individuals high in hostility.

## Figures and Tables

**Table 1:** The pearson correlations.

Variable	PSQI	DSQ	CogHo	BHo	PSS
PSQI					
DSQ	.282*				
CogHo	−.421**	−.260*			
BHo	−.050	−.042	.208**		
PSS	−.584***	−.515***	.352*	−.104	
SPE	−.432***	−.517***	.321*	−.025	.613***

*N* = 66. PSQI: Pittsburg Sleep Quality Index; DSQ: daily sleep quality; CogHo: cognitive hostility subscales; BHo: aggressive responding subscale; PSS: perceived stress scale; SPE: stress per event.

*P < .05, **P < .01, ***P < .001.

**Table 2:** Cognitive hostility and daily SQ mediated by stress per event.

Step	IV	DV	*B*	SE	*Β*	*R* ^2^	Adj *R* ^2^	sr^2^
1	CogHo	DSQ	−.092	.043	−.290	.068	.053	.053*
2	CogHo	SPE	.092	.034	.321	.103	.089	.089*
3	CogHo	DSQ	−.037	.040	−.105	.277	.254	.010
	SPE	DSQ	−.596	.140	−.483	.277	.254	.210*
			Standardized indirect effect = −.0550, *P* < .001					
			95% CI = −.108; −.013					

*N* = 66. SPE: average stress severity rating per event; DSQ: daily sleep quality, CogHo: cognitive hostility.

*P < .05.

**Table 3:** Cognitive hostility and the PSQI mediated by PSS.

Step	IV	DV	*B*	SE	*β*	*R* ^2^	Adj *R* ^2^	sr^2^
1	CogHo	PSQI	.388	.105	.421	.177	.164	.177*
2	CogHo	PSS	.718	.239	.352	.124	.110	.124*
3	CogHo	PSQI	.227	.097	.246	.393	.374	.053*
	PSS	PSQI	.225	.047	.497	.393	.374	.216*
			Standardized indirect effect = .1613, *P* < .001					
			95% CI = .072; .279					

PSS: Perceived Stress Scale; PSQI: Pittsburg Sleep Quality Index, CogHo: cognitive hostility.

*P < .05.
